# Predictive value of IgE/IgG_4_ antibody ratio in children with egg allergy

**DOI:** 10.1186/1710-1492-8-9

**Published:** 2012-06-07

**Authors:** Shindou Okamoto, Shoichiro Taniuchi, Kyoko Sudo, Yasuko Hatano, Keiji Nakano, Tomohiko Shimo, Kazunari Kaneko

**Affiliations:** 1 Department of Pediatrics, Kansai Medical University, Fumizonocho 10-15, Moriguchi, Osaka, 570-8506, Japan

**Keywords:** Food allergy, IgG_4_, egg white, IgE, Food challenge

## Abstract

**Background:**

The aim of this study was to investigate the role of specific IgG_4_ antibodies to hen’s egg white and determine their utility as a marker for the outcome of oral challenge test in children sensitized to hen’s egg

**Methods:**

The hen’s egg oral food challenge test was performed in 105 sensitized children without atopic dermatitis, and the titers of egg white-specific immunoglobulin G_4_ (IgG_4_) and immunoglobulin E (IgE) antibodies were measured. To set the cut-off values of IgG_4_, IgE, and the IgE/IgG_4_ ratio for predicting positive results in oral challenges, receiver operating characteristic curves were plotted and the area under the curves (AUC) were calculated.

**Results:**

Sixty-four of 105 oral challenges with whole eggs were assessed as positive. The AUC for IgE, IgG_4_, and IgE/IgG_4_ for the prediction of positive results were 0.609, 0.724, and 0.847, respectively. Thus, the IgE/IgG_4_ ratio generated significantly higher specificity, sensitivity, positive predictive value (%), and negative predictive value (%) than the individual IgE and IgG_4_. The negative predictive value of the IgE/IgG_4_ ratio was 90% at a value of 1.

**Conclusions:**

We have demonstrated that the egg white-specific serum IgE/IgG_4_ ratio is important for predicting reactivity to egg during food challenges.

## Background

Hen’s egg is one of the common food in children, and is the most common immediate-type food allergy in Japanese children [1]. The proteins contained in hen’s eggs are, however, important sources of nutrients during childhood. Therefore, a risk of accidental exposure to antigenic proteins is higher in children sensitized to hen’s eggs than in children sensitized to other foods, such as fish [2,3]. Accordingly, an accurate diagnosis of food allergies is warranted with a proper history correlated to immunoglobulin E (IgE)-mediated reactions (skin prick tests and/or antigen-specific IgE in the serum) and food challenge tests that may induce an anaphylactic reaction. To optimize the utility of food challenge tests, Sampson et al. [4] proposed cut-off levels of allergen-specific IgE for predicting clinical allergic reactivity to egg, milk, peanut, and fish. These initial studies were followed by other studies that identified a large variety of cut-off levels [5-7] but those were largely dependent on age and the type of food-induced reactions (e.g., gastrointestinal symptoms or skin reactions).

Immunoglobulin G (IgG) antibodies to food allergens are produced in both atopic and non-atopic children. IgG antibody responses to food allergens are observed at birth and are maternally derived. The production of IgG antibodies to food allergens peaks in early childhood and declines by 8 years of age [8,9]. The presence of IgG_4_ antibodies is commonly identified in atopic patients but their role remains unclear [10].

The mechanism underlying the regulation of IgE and IgG_4_ production is controversial. Interleukin (IL)-4 from Th2 cells induces both IgE and IgG_4_ switching in B cells [11], while IL-10 inhibits IgE production but upregulates the secretion of IgG_4_, suggesting different ways of controlling IgE and IgG_4_ production [12]. The mechanism(s) of tolerance induction to allergens also remains unknown, but it has been proposed that exposure to some airborne allergens may favor immunological tolerance development via a modified Th2 response characterized by a high IgG_4_/IgE ratio [13]. While information regarding food allergens is scarce in the literature, several studies show a relationship between oral tolerance and increased specific IgG_4_ level [14-18]. It is clear that oral immunotherapy induces an increase in specific IgG_4_ levels [14,15,18]. However, the role of specific IgG_4_ to food allergens remains controversial in the natural course of food allergy.

Based on these findings, this study was undertaken to investigate the role of egg white-specific IgG_4_ antibodies and determine whether these antibodies could be used as a marker for the outcome of oral challenge tests in children sensitized to hen’s eggs.

## Patients and methods

### Patients

Children, sensitized to egg and egg products, with symptoms relating to the skin and/or respiratory and gastrointestinal tracts, and referred to Kansai Medical University Takii Hospital were consecutively included in the study. We retrospectively reviewed the clinical data of 105 children (41 females and 64 males) who exhibited immediate, IgE-mediated allergy to hen’s egg and received oral challenge tests at our clinic between January 2007 and December 2009. Patients with atopic dermatitis were excluded from the study, because of the following reasons: (1) if the enrolled subjects had atopic dermatitis, conducting the food challenge test would be difficult owing to the presence of eczema before the food challenge test, and (2) increased total IgG_4_ and specific IgG_4_ levels may be expected in the patients with atopic dermatitis with no regard to tolerance of egg allergy [10]. The median age of patients was 5.0 years (range, 12 months-13 years).

## Methods

Serum samples were obtained from all subjects on the day of the food challenge test and immediately processed for quantification of egg white-specific IgE antibody titers using the UniCAP SystemTM (Phadia, Uppsala, Sweden), according to the manufacturer's instructions. Egg white-specific IgG_4_ antibody levels were measured using the ImmunoCAP ISAC® assay kit IgG_4_ ((Phadia, Uppsala, Sweden). Serum samples were stored for −70°C and examined within 6 months after the day of food challenge. Briefly, egg white-specific IgG_4_ antibodies were measured in serum samples using the Phadia 250 instrument (Phadia, Uppsala, Sweden), according to the manufacturer’s instructions. The method comprised the following steps. The test serum was added to the ImmunoCAP (a solid phase with covalently bound egg white protein) and incubated. After washing to eliminate any nonspecific IgG_4_, enzyme-labeled anti-IgG_4_ was added to form a complex. After another incubation of 150 min, any unbound labeled anti-IgG_4_ antibodies were eliminated by washing. The labeled allergen-antibody complex was then incubated with a developing agent and, after stopping the reaction, the fluorescence of the eluate was measured by fluorimetry (FluoroCount). Specific antibody concentrations are expressed in percentage ([fluorescence of sample 1/fluorescence of reference serum] × 100). The higher the response, the more specific IgG_4_ is present in the specimen. To evaluate the test results, the responses from patient samples were directly compared with a reference serum run in parallel. ImmunoCAP Specific IgG_4_ Calibrators were used for total IgG_4_ determination, and values are expressed in μg/L. In the ImmunoCAP Specific IgG_4_ assay, these calibrators were used for the determination of specific IgG_4_ antibodies, and values are expressed in mg_A_/L, where A represents antigen-specific antibodies. Values above the limit of quantitation represent a progressive increase in the concentration of antigen-specific IgG_4_ antibodies. With a sample dilution of 1:100, IgG_4_ antibody levels up to 30 mgA/L were measured. The crossreactivity of the enzyme-labeled anti-IgG_4_ with IgG_1_, IgG_2_, IgG_3_, IgA, IgM, and IgE was <0.5%.

### Oral food challenges

All egg-food challenges were open challenges, performed in hospital settings and supervised by physicians. Clinical features to react hen’s egg were investigated for clinical purposes via an open challenge test as described in the Japanese food allergy management guideline 2008 [2]. A double-blind placebo-control food challenge (DBPCFC) is the gold standard for clinical studies, but is a time-consuming test for general practice. We could not assess the subjective symptoms by the open challenge test. Therefore, if the patients had subjective allergic symptoms such as nausea, abdominal pain, sore throat, or itching, we increased the loading dose before the objective symptoms appeared. During the challenge, full emergency equipment was at hand. The children’s parents prior to enrolment in the study gave informed consent. Patients taking anti-histamines were asked to avoid them for at least 48 h before the challenge, but topical steroids were allowed. Patients were admitted to our day clinic in the morning in a fasting state. Challenge material for open challenges was cooked egg boiled for 3 min and then steamed for 10 min. The initial challenge dose and the following doses were set according to the history of the last reaction, but were similar in most patients (eggs: 0.8, 1.6, 3.2, 6.4, 12.8, and 25.6 g). When the patients tolerated the first dose, the following one was given every 30 min. When a reaction to a very low dose was suspected, the first challenge dose was 0.4 g. The doses and the time interval between 2 doses were adapted. The challenge was interrupted if children demonstrated unambiguous clinical reactivity [11] or after the administration of 50.4 g of egg. All children were then observed for at least 3 more hours after the end of the feeding. If a child exhibited obvious allergic symptoms, such as rash, coughing, vomiting, or diarrhea, to hen’s egg under loading doses of less than 25.6 g, he/she was considered to have positivity to hen’s egg. Otherwise, they were considered negative.

### Statistical methods

The Mann–Whitney test, the χ2 test (2-tailed), or the Kruskal-Wallis rank test was used to test differences between groups. A p-value of 0.05 was considered significant. Performance characteristics (i.e., sensitivity and specificity) were calculated for various cut-off values, including the optimal cut-off values proposed by the receiver operating characteristics (ROC) plots. Statistical analysis was carried out using SAS System V8.2 (SAS Institute Inc., Cary, NC, USA). The box plot and whisker plots were generated in the study because the data did not show normal distribution.

## Results

### Oral food challenge results

Oral food challenges using egg white were performed in 105 children sensitized to hen’s eggs. Forty-five children were negative, while 60 were assessed positive: 25 children reacted under loading doses of <0.8 g of hen’s egg, 13 at 1.6 g, 11 at 3.2 g, and 11 at 6.4 g. No child reacted at doses of either 12.8 g or 25.6 g.

### Summarized clinical data of the positive and negative groups

Patient characteristics are presented in Table 1. Patients in the positive food challenge (PFC) group and the negative food challenge (NFC) group exhibited similar history of clinical symptoms, except for the frequency of anaphylaxis. Sixteen and 4 patients in the positive and negative groups, respectively, experienced food-induced anaphylaxis (p < 0.05)

**Table 1:** Clinical data of the negative food challenge (NFC) and positive food challenge (PFC) groups in the challenge test

	NFC	PFC	p value
Number of patients	45	60	
Age (y), median (range)	4 (1–13)	5 (1–12)	0.06389*
Male/female (n)	30/15	34/26	0.4024*
Frequency of allergic symptoms at accidental ingestion			
Once (n)	35	37	0.067**
2–10 times (n)	3	14	
10 times (n)	7	9	
Frequency of anaphylaxis at accidental ingestion (n)	4/45	16/60	0.025*
Duration since last episode (y; mean [SD])	3.2 (2.2)	3.4 (1.7)	0.3162*
Condition of elimination			
Complete	38	51	0.844*
Partial^#^	7	9	

*, The χ2 test (2-tailed) was used; **, The Kruskal-Wallis rank test was used; ***, The Mann–Whitney U test (2-tailed) was used.

#: After the patients ate egg products or less than 1 g of heated eggs, complete elimination was performed before the challenge test.

### Titers of egg white-specific IgE, IgG_4_, and IgE/IgG_4_ antibodies

**Figure 1:**
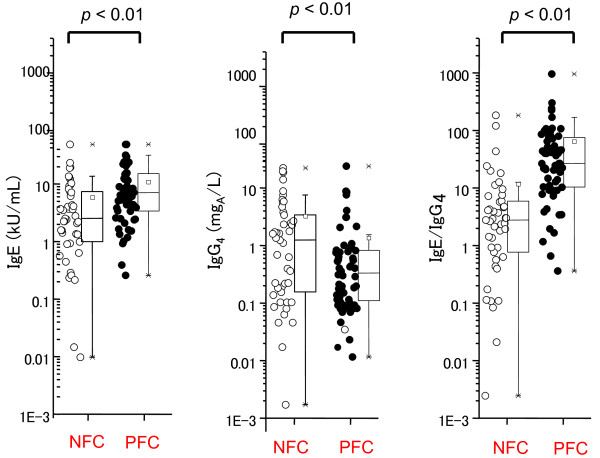
**Dot plots and Whisker’s box plots showing levels of specific IgE (left column), IgG**_**4**_**(middle column), and IgE/IgG**_**4**_**ratio (right column) to determine the NFC (negative food challenge) or PFC (positive food challenge) group of egg allergy.**

The median values of the concentrations of egg-specific IgE antibodies were 5.15 kUA/L (2.03-15.10 kUA/L; 25-75% observation) in the NFC group (45 children) and 14.45 kUA/L (6.93-30.9 kUA/L) in the PFC group (60 children; p < 0.01; Figure 1). The median values of the concentrations of egg-specific IgG_4_ antibodies were 2.17 mgA/L (0.27-5.89 mgA/L; 25-75% observation) and 0.29 mgA/L (0.095-0.715 mgA/L) in the NFC and PFC groups, respectively (p < 0.01; Figure 1). In the PFC group, the ratio of egg-specific IgE/IgG_4_ antibodies (IgE/IgG_4_ ratio) was 4.83 (1.34-10.37; 25-75% observation), whereas it was 46.71 (18.05-131.04) in the NFC group (p < 0.01; Figure 1).

### Ratio of egg-specific IgE/IgG_4_ antibodies for each dosage of eggs in the challenge test

**Figure 2:**
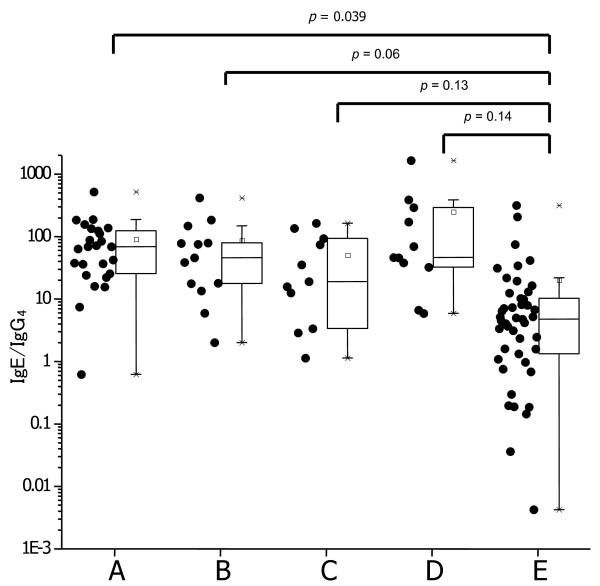
**Dot plots and Whisker’s box plots for IgE/IgG**_**4**_**ratios in A (reacts at less than 0.8 g of total eggs), B (1.6 g), C (3.2 g), D (6.4 g), and E (NFC) groups of egg allergy.**

The enrolled subjects who received the hen’s egg oral challenge test were divided into 5 groups based on their reactivity to the dosages of hen’s eggs in the challenge test. The A group (n = 25) reacted at less than 0.8 g of eggs, the B group (n = 13) at 1.6 g, the C group (n = 11) at 3.2 g, and the D group (n = 11) at 6.4 g. The E group was negative. The IgE/IgG_4_ ratio in the NFC group was significantly lower than those of the other groups, except the A group (Figure 2; p < 0.05). The IgE/IgG_4_ ratio also gradually decreased with decreasing reactivity (Figure 2).

### IgE, IgG_4_, and, IgE/IgG_4_ cut-off levels for predicting the outcome of the hen’s egg white oral challenge test

**Figure 3:**
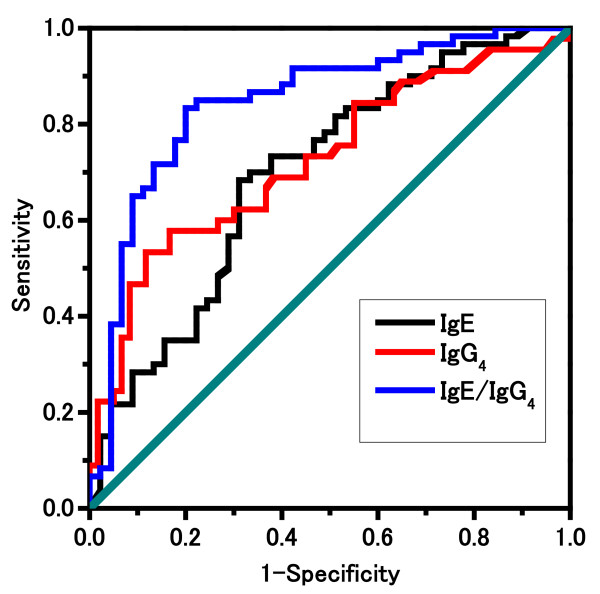
**Receiver operating characteristic (ROC) curve for IgE (black line), IgG**_**4**_**(red line), and IgE/IgG**_**4**_**(blue line) to determine the positivity or negativity of egg allergy**. The ROC curve for IgE/IgG_4_ is superior to those for IgE and IgG_4_ levels. The AUCs for IgE, IgG_4_, and IgE/IgG_4_ for the prediction of positive results were 0.609, 0.724, and 0.847, respectively.

To set the cut-off values of IgG_4_, IgE, and the IgE/IgG_4_ ratio for predicting positive results in oral challenges, ROCs were plotted and the AUCs were calculated. The AUC for serum egg-specific IgE was 0.697; for the estimated cut-off of 8.9 IU/mL, values of 68.3% and 68.9% were obtained for the sensitivity and specificity of the assay, respectively (Figure 3, Table 2). The positive predictive value (PPV) and negative predictive value (NPV) were 74.5% and 62%, respectively (Table 2). The AUC for serum egg-specific IgG_4_ was 0.724; for the estimated cut-off of 1.93 mgA/L, values of 53.3% and 88.3% were obtained for the sensitivity and specificity of the assay, respectively (Figure 3, Table 2). The PPV and NPV were 77.4% and 71.4%, respectively (Table 2). In contrast, the AUC for the egg-specific IgE/IgG_4_ ratio was 0.847; for the estimated cut-off of 13.63, the sensitivity and specificity of the assay were 83.3% and 80%, respectively (Figure 3, Table 2). The PPV and NPV were 84.7% and 78.3%, respectively (Table 2).

**Table 2:** Cut-off values, sensitivity, specificity, and AUC* for egg-specific IgE, IgG _**4 **_**, and IgE/IgG **_**4 **_

	IgE	IgG_4_	IgE/IgG_4_
Cut-off value	8.9 (kUA/L)	1.93 (mg_A_/L)	13.63
Sensitivity (%)	68.3	53.3	83.3
Specificity (%)	68.9	88.3	80.0
Positive predictive value (%)	74.5	77.4	84.7
Negative predictive value (%)	62	71.4	78.3
AUC* (%)	0.697	0.724	0.847

*Area under the curve.

**Figure 4:**
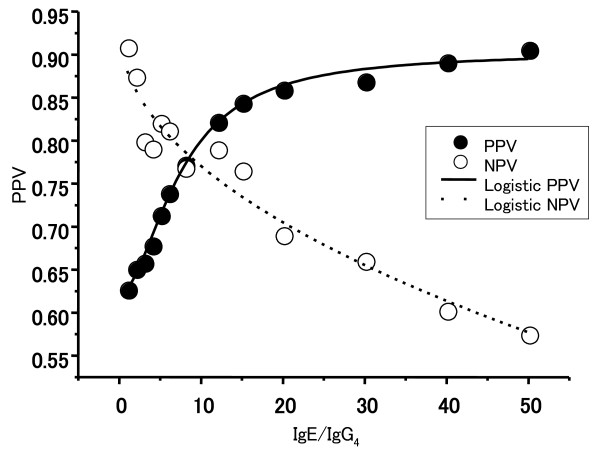
**Probability of reacting to egg at a given egg-specific IgE/IgG**_**4**_**ratio.**

As the AUC for IgE/IgG_4_ ratio was higher than those for individual IgE and IgG_4_ values, PPV and NPV curves of the egg IgE/IgG_4_ ratios were calculated as shown in Figure 4; the PPV was 90% at a value of 50 and the NPV was 90% at a value of 1.

### Comparison of AUCs between the over 5 years of age and under 4 years of age groups for predicting the outcome of the hen’s egg white oral challenge test

The patients were divided into 2 groups, over 5 years of age and under 4 years of age, and their ROC curves were compared. The area under the ROC curves (AUCs) for egg-specific IgE, IgG_4_, and IgE/IgG_4_ in the older age group were 0.638, 0.802, and 0.861, respectively. In the younger group, the AUCs for egg-specific IgE, IgG_4_, and IgE/IgG_4_ were 0.708, 0.655, and 0.832, respectively. In the older group, the AUC for IgG_4_ was especially high compared to that in the younger group (0.802 vs. 0.655). All 4 parameters exhibited relatively high IgE/IgG_4_ ratios compared to IgE and IgG_4_ in the 2 groups.

## Discussion

In recent years, it has been highly recommended that unclear cases of food allergy should be confirmed by standardized food challenges, such as the DBPCFC, for definite diagnosis although it carries a risk of severe adverse events, such as anaphylaxis [19,20]. In the present study, we demonstrate for the first time that the measurement of serum IgG_4_ antibodies as well as IgE antibodies to hen’s egg white is of value in the prediction of reactivity to hen’s egg during food challenges, and therefore helpful in the confirmation of food tolerance in egg-sensitized children. The titers of food-specific IgE antibodies have already been reported to be useful in the prediction and possible diagnosis of IgE-mediated food allergy; several studies have proposed “cut-off” values of antigen-specific serum IgE or weal diameter sizes for the diagnosis of food allergy, to optimize the use of food challenge tests [21,22]. Sampson and Ho identified PPV and NPV values of specific IgE antibodies against different foods, such as milk, egg, and peanut, using the CAP-FEIA system [4]. They found that, in the population mainly referred for severe atopic dermatitis, egg-specific IgE cut-off levels for >95% PPV, >90% PPV, and >90% NPV were 6 kUA/L, 2 kUA/L, and 0.6 kUA/L, respectively. This study has been followed by others, although the PPV, NPV, and cut-off values varied considerably [23-25]. Compared to these studies, the PPV and NPV of IgE to egg white in our study were relatively low. Both the sensitivity and AUC for egg white-specific IgE in our study were also low compared to 2 earlier studies [23,24]. These differences may have arisen because the patients with atopic dermatitis were excluded in our study group or because the enrolled studied population was relatively older than that of previous studies. Whatever the reason, we feel that a more sensitive and more specific marker is clearly needed for the prediction of reactivity to hen’s egg during food challenges.

Recent studies on immunotherapy with inhalant allergens suggest a protective role of IgG_4_ antibodies during treatment [26,27]. It has been suggested that allergen-specific IgG_4_ antibodies act as blocking antibodies by competing with IgE for allergen binding to IgE receptor-expressing cells, such as mast cells and basophils [11], and competition between IgE and IgG_4_ antibodies at the level of antigen-presenting cells has also been detected *in vitro*[26]. The production of both IgE and IgG_4_ antibodies is up-regulated by IL-4 produced from activated Th2 cells [11]. However, IL-10 secreted by regulatory T cells during immunotherapy potentially suppresses IgE production and simultaneously increases IgG_4_ production [28]. Another immune response with a protective effect against the development of allergic disease is a modified Th2 immune response that includes high levels of IgG_4_ antibodies in the absence of IgE antibodies [29].

Several reports have described the role of specific IgG_4_ in natural tolerance development in food allergy. Stapel et al. have shown that food-specific IgG_4_ does not indicate food allergy or intolerance, but rather indicates immunological tolerance, linked to the activity of regulatory T cells [29]. Ruiter et al. [30] have reported similar results in cow’s milk allergy showing that IgG_4_ levels were the highest in atopic subjects who were tolerant to cow’s milk, whereas Shek et al. [31] have described an increased IgG_4_ response in children allergic to cow’s milk compared to atopic patients without suspected food allergy. Ahrens et al. [32] reported no difference in hen’s egg white-specific IgG_4_ levels between tolerant and allergic children. The discrepancy between our study and Ahrens’s result may be due to the following reasons: (1) the age of the study population, (2) the challenged materials, and (3) the procedure of the challenge test, (4) the exclusion of atopic dermatitis patients. The main reason for the discrepancy may be the age of the population. The mean age in our study was 5 years, although it shows 2 years of age of its study. We also observed a lower level of AUC for hen’s egg white-specific IgG_4_ in the under 5 years of age group than in the over 5 years of age group. Children younger than 2 years of age have less ability to produce IgG_4_[33]. Thus, it remains unclear as to whether the role of IgG_4_ in food allergy is related to tolerance or repeated exposure to food ingestion. In our study, children demonstrating a lower ratio of IgE/IgG_4_ to hen’s egg (i.e., relatively higher IgG_4_ to IgE) were more likely to be able to eat eggs. The cut-off level of IgE/IgG_4_ for more than 90% of the NPVs was 1, and that for 20% of the post-test probability values was 13.5, based on the calculation of negative likelihood ratios (0.2). This means that 90% and 80% of patients who showed cut-off levels of IgE/IgG_4_ that were less than 1 and 13.5, respectively, were able to eat hen’s eggs.

## Conclusions

Our study demonstrated for the first time that the IgE/IgG_4_ ratio to egg white is a more useful parameter for predicting the outcome of oral challenge with eggs than IgE alone in patients without atopic dermatitis or particularly in older children. Further study will be needed to evaluate the role of egg white-specific IgG_4_ in oral tolerance induction in hen’s egg allergy.

## Competing interests

The authors declare that they have no competing interests.

## Authors’ contributions

SO and ST conceived the study, designed the study and wrote the paper. SK and YH participated in its design and writing. NK and TS helped to edit the manuscript. KK helped to collect data for analysis. All authors read and approved the final manuscript.
