# Correction: Predictive value of IgE/IgG4 antibody ratio in children with egg allergy

**DOI:** 10.1186/1710-1492-9-34

**Published:** 2013-09-09

**Authors:** Shindou Okamoto, Shoichiro Taniuchi, Kyoko Sudo, Yasuko Hatano, Keiji Nakano, Tomohiko Shimo, Kazunari Kaneko

**Affiliations:** 1 Department of Pediatrics, Kansai Medical University, Fumizonocho 10-15, Moriguchi, Osaka 570-8506, Japan

## Correction

Following the publication of our article [1] two separate errors within the manuscript have come to our attention. These errors can both be found in the *Results* section.

Firstly, in the *Titers of egg white-specific IgE, IgG, and IgE/IgG antibodies* subsection, the last sentence stated: “In the PFC, the ratio of egg-specific IgE/IgG4 antibodies (IgE/IgG4) was 4.83 (1.34-10.37; 25-75% observation), whereas it was 46.71 (18.05-131.04) in the NFC group (p<0.01; Figure one)”.

It should instead read “In the NFC, the ratio of egg-specific IgE/IgG4 antibodies (IgE/IgG4) was 4.83 (1.34-10.37; 25-75% observation), whereas it was 46.71(18.05-131.04) in the PFC group (P<0.01; Figure one)”.

Secondly, in the *Ratio of egg-specific IgE/IgG antibodies for each dosage of eggs in the challenge test* subsection, the 4th sentence stated: “The IgE/IgG4 ratio in the NFC group was significantly lower than those of the other groups, except the A group (Figure two; p<0.05)”.

It should instead read: “The IgE/IgG4 ratio in the NFC group was not significantly those of the other groups, except the A group than (Figure two; P<0.05)”.

Please note that the conclusions made in the original publication were based on the correct data, and as such they are unaffected by the errors made.
